# Evidence for a useful life of more than three years for a polyester-based long-lasting insecticidal mosquito net in Western Uganda

**DOI:** 10.1186/1475-2875-10-299

**Published:** 2011-10-13

**Authors:** Albert Kilian, Wilson Byamukama, Olivier Pigeon, John Gimnig, Francis Atieli, Lizette Koekemoer, Natacha Protopopoff

**Affiliations:** 1 Malaria Consortium - International, London, UK; 2 Malaria Consortium - Africa Regional Office, Kampala, Uganda; 3 Walloon Agricultural Research Centre (CRA-W), Agriculture and Natural Environment Department, Gembloux, Belgium; 4 Division of Parasitic Diseases, Centers for Disease Control and Prevention, Atlanta, USA; 5 Centre for Vector Biology and Control Research, Kenya Medical Research Institute, Kisumu, Kenya; 6 Vector Control Reference Unit, National Institute for Communicable Diseases of the NHLS, Private Bag X4, Sandringham; 7 Malaria Entomology Research Unit, School of Pathology, University of the Witwatersrand, Johannesburg 2000, South Africa

## Abstract

**Background:**

Long-lasting insecticidal nets (LLIN) are now standard for the prevention of malaria. However, only products with recommendation for public use from the World Health Organization should be used and this evaluation includes the assessment of net effectiveness after three years of field use. Results for one of the polyester-based products, Interceptor^® ^is presented.

**Methods:**

In five villages, 190 LLIN and 90 nets conventionally treated with the insecticide alpha-cypermethrin at 25 mg/m^2 ^were distributed randomly and used by the families. Following a baseline household survey a net survey was carried out every six months to capture use, washing habits and physical condition of the nets. Randomly selected nets were collected after 6, 12, 24, 36 and 42 months and tested for remaining insecticide content and ability to knock-down and kill malaria transmitting mosquitoes.

**Results:**

During the three and a half years of observation only 16 nets were lost to follow-up resulting in an estimated attrition rate of 12% after three and 20/% after 3.5 years. Nets were used regularly and washed on average 1.5 times per year. After three and a half years 29% of the nets were still in good condition while 13% were seriously torn with no difference between the LLIN and control nets. The conventionally treated nets quickly lost insecticide and after 24 months only 7% of the original dose remained (1.6 mg/m^2^). Baseline median concentration of alpha-cypermethrin for LLIN was 194.5 mg/m^2 ^or 97% of the target dose with between and within net variation of 11% and 4% respectively (relative standard deviation). On the LLIN 73.8 mg/m^2 ^alpha-cypermethrin remained after three years of use and 56.2 mg/m^2 ^after three and a half and 94% and 81% of the LLIN still had > 15 mg/m^2 ^left respectively. Optimal effectiveness in bio-assays (≥95% 60 minute knock-down or ≥ 80% 24 hour mortality) was found in 83% of the sampled LLIN after three and 71% after three and a half years.

**Conclusions:**

Under conditions in Western Uganda the tested long-lasting insecticidal net Interceptor^® ^fulfilled the criteria for phase III of WHO evaluations and, based on preliminary criteria of the useful life, this product is estimated to last on average between three and four years.

## Background

The technology of long-lasting insecticidal nets (LLIN) was developed in the late 1990'ties as a response to the poor re-treatment practices for conventionally-treated mosquito nets [1] and the first evaluation reports for a polyethylene-based LLIN was published in 1999 [2] followed only three years later by one for a polyester-based LLIN [3]. Since then LLIN have become the recommended approach for malaria prevention with mosquito nets [4] and in some countries the proportion of all nets that are LLIN is already exceeding 90% [5].

There are a number products on the market that use the term "long-lasting" to advertise their insecticide-treated net product but not all of these are actually LLIN. Criteria for use of public funds on the purchase of LLIN as practiced by all major funders is the recommendation from the WHO Pesticide Evaluation Scheme (WHOPES) that a LLIN brand is suitable for malaria prevention. The evaluation of specific products by WHOPES comprises three phases of testing. Phase I consists of laboratory testing of wash resistance and insecticide regeneration on the surface of the net. This is followed by small-scale field trials usually using experimental huts to test wash-resistance and efficacy in phase II. Finally large-scale field trials under "real life conditions" are done in phase III testing the long-lasting efficacy, community acceptance and safety observations [6]. If a product has fulfilled the testing criteria of phase I and II of resisting at least 20 WHO standard washes it usually receives an interim recommendation while full recommendation is given after it has been shown to remain effective for at least three years of field use during phase III. Currently there are three LLIN brands which have full recommendation for public health use and nine with interim recommendations [7]. One of the latter is the Interceptor^® ^brand, a polyester based LLIN using the coating technology where a resin based polymer coating is used as the insecticide reservoir for replacement of surface insecticide and this coating is bound to the surface of the polyester filament. This LLIN received interim WHOPES recommendation in December 2006 [8] and field studies so far show a high level of acceptability and promising effectiveness after up to one year of follow-up [9-11].

This study presents the results of three and a half years of field testing of the Interceptor^® ^LLIN brand in Western Uganda in a setting where other LLIN brands have been or are being tested allowing a direct comparison of the performances.

## Methods

### Study design and area

The general design was a prospective study with the single net as the unit of observation and multiple cross-sectional surveys for evaluation of the primary outcomes, bio-assay and chemical residue analysis. The study protocol followed the WHO guidelines for phase III field trials [6] with minor modifications and compared the performance of the LLIN with that of a comparable mosquito net conventionally treated with the same insecticide as used in the LLIN. Study site was five villages in Kirongo Parish, Kyenjojo District, which have been described in detail previously [12]. In short, this is an rural area in Western Uganda with moderate climate at altitudes of 1350-1550 m and a meso- to hyperendemic malaria situation.

### Nets and net treatment

The LLIN Interceptor^® ^was provided by BASF Corporation (Research Triangle Park, NC, USA). They were white, rectangular multifilament polyester nets of 75 denier and medium size (160 × 150 × 180 cm W × H × L). Long-lasting treatment was applied at production with FENDOZIN^®^, a mixture of the insecticide alpha-cypermethrin with a binding polymer at a target dose for the insecticide of 6.7 g/kg or 200 mg/m^2 ^[13].

Nets for the conventional treatment were also white, rectangular multifilament polyester nets of 75 denier (Siamdutch Mosquito Netting Co., Bangkok, Thailand) but of size 190 × 150 × 180 cm (W × H × L). Net treatment was done by a team of trained dippers and supervised by one of the authors (AK). Nets were treated individually in basins using one sachet of 6 ml alpha-cypermethrin 6% (FENDONA^®^, BASF, Midrand, South Africa) and a standard amount of water adequate for the size of net. Based on the content of the sachet of 360 mg of alpha-cypermethrin and the average size of the nets of 14.1 m^2 ^the target dose was 24.9 mg/m^2^. Nets were dried lying flat on the ground without direct exposure to sunlight. The dipping team was provided with adequate protective gear.

### Net distribution

A total of 200 LLIN and 100 conventionally treated nets were provided or prepared.

After treatment 10 of the conventionally treated nets were randomly selected for the baseline assessment. Similarly, 10 of the LLIN were also selected as baseline nets at this time. All nets for distribution to households (190 LLIN, 90 conventionally treated) were identified with a unique ID number written with wash resistant. The allocation of numbers to nets was random and only the principal investigator had the allocation list. In addition, each net was also marked with a water-soluble ink as a quality control for the assessment of washing.

From previous and on-going studies a complete household list for all 5 villages was available indicating the number of beds in the household and any study net already allocated. Based on these lists study nets were randomly allocated to households by the village health workers. The net allocation list was computerized and served as a net master list. Net allocation took place in May 2006.

### Surveys

A survey assessing the demographic and socio-economic characteristics of all households participating in the LLIN studies was undertaken in May 2006 before net distribution.

Net follow-up surveys were then undertaken every six months in September or October and April or May with a total of eight surveys, the last being in April 2010. During the net surveys all remaining nets were assessed for usage, dirtiness (clean, slightly dirty, dirty or very dirty as subjectively judged by the interviewer), washing frequency during the past six months, method of washing and drying as well as number and size of any holes in the net. Holes were categorized in three groups:

Size 1 (finger size): Any hole not larger than 2 cm in maximum diameter

Size 2 (hand size): Any hole larger than 2 cm and less than 10 cm in maximum diameter

Size 3 (head size): Any hole larger than 10 cm in maximum diameter

Any loss of nets was also recorded and the net master list updated accordingly.

### Net collections and sample preparation

From the master list nets were randomly selected for outcome evaluation with 2-3 replacement numbers drawn in case the selected nets could not be traced on the day of sampling. These lists were communicated to the field team and nets were collected after the net follow-up surveys in order to ensure that washing information for the collected net was obtained. Households received a new LLIN as replacement but these nets were not part of the study. LLIN collections were done after six, 12, 24, 36 and 42 months of follow-up with a target sample size of 40 nets each except for the sample after 42 months. As the study was originally only planned for 36 months this collection comprised of all remaining LLIN, which was 21. Conventionally treated nets were sampled at six and 12 months with a target of 40 nets each and then all remaining nets at month 24.

Sampled nets were prepared in the laboratory in the following way: each net was carefully inspected and the general condition, presence of the wash-control mark and number and sizes of holes recorded. Using specially prepared templates netting material was cut always from the same location on the net, i.e. on the long side of the net below the label and half way between roof and lower border. The size of the sample was 30 × 30 cm for bio-assay samples and 10 × 10 cm for chemical residue samples. The chemical residue sample was cut directly next to the bio-assay sample. The labelled samples were packed in aluminium foil and kept in a fridge at 4-8°C before transport to the laboratory.

For each net one sample was taken for each laboratory test except for the baseline nets for which one bio-assay and three chemical residue samples were obtained. The chemical residue samples were taken from different sides of the net to allow assessment of within-net variability of insecticide distribution.

### Bio-assays

Bio-assays were carried out by the Centers of Disease Control, Atlanta, USA using WHO standardized procedures. For the tests 2-4 day old, unfed female *Anopheles gambiae s.s*. (Kisumu strain) were used. This species has been well established in culture for a long time and is known to be pyrethroid sensitive. Five mosquitoes were introduced into WHO cones at a time and four cones applied simultaneously onto the net sample with a three-minute exposure of the vectors. Tests were made at 25 ± 2°C under subdued light. After exposure, females were grouped into batches of 10 or 20 in 200 mL plastic cups and maintained at 28°C ± 2°C and 80% ± 10% relative humidity with honey solution provided. For each sample, a total of 40 mosquitoes were used. For each series a control was run with no exposure and results were only used if control mortality was less than 5%. Numbers of mosquitoes knocked down were recorded at 30 and 60 minutes and knock down rate at 60 minutes (KD60) calculated. Percentage mortalities were recorded after 24 hours using immediate and delayed mortality as defined by WHO guidelines [6], i.e. mosquitoes were scored as dead if they could not fly or stand upright on either the side or the bottom of the paper cups. Those that had lost one or more legs and could fly and stand upright without collapsing were considered to be alive.

In May 2009, the testing was shifted to the CDC partner Kenya Medical Research Institute in Kisumu using the same mosquito strain and methodology. However, due to the development of a resistance problem of the Kisumu strain the last bio-assays (42 months) were done at the Vector Control Reference Unit, National Institute for Communicable Diseases, Johannesburg, South Africa using the *An. gambiae s.s*. SUA strain. The methodology differed in that the WHO tubes for vector sensitivity testing were used as exposure device introducing the netting instead of the paper. Otherwise conditions were the same as in the CDC tests.

### Chemical residue

Chemical residue analysis was done at the Wallon Agricultural Research Centre (CRA-W), Gembloux, Belgium (WHO Collaborating Centre for Quality Control of Pesticides) using the ISO 17025 accredited analytical method RESMM002. The samples were measured and weighed and then introduced into a 100 mL Erlenmeyer flask. Alpha-cypermethrin was extracted from the sample by heating under reflux for 60 minutes with 40 mL xylene. After cooling to ambient temperature the extract was quantitatively transferred into a 50 mL volumetric flask. The flask was filled up to volume with xylene. A 10 times dilution was achieved in xylene. The final extract was then analysed for determination of alpha-cypermethrin by Capillary Gas Chromatography with ^63^Ni Electron Capture Detection (GC-ECD) using an external standard calibration. For each sample two chromatographic injections were performed and the mean reported as g/kg and mg/m^2 ^of alpha-cypermethin. Before the analysis of samples, the analytical method was successfully validated on its specificity, linearity of detector response, repeatability, accuracy and limit of quantification. During the analysis of samples, the performance of the analytical method was checked in order to validate the analytical results.

### Data entry and analysis

All data was entered in an EpiData 3.1 data and then transferred to Stata 11.0 statistical software (Stata Corp., College Station, USA) for management and analysis. For proportions (rates) exact binomial 95% confidence intervals were used. For continuous variables either the arithmetic or geometric mean or median was used depending on the distribution of values compared to a normal distribution. For the core outcomes multivariate analysis was applied using a logit or linear regression model with all potential co-variates. For analysis of net survey results with repeated observations on the same net generalized estimation equations (gee) were used.

The primary outcome of net effectiveness was based on the bio-assay results using the following criteria:

Optimal effectiveness: KD60 ≥ 95% or functional mortality ≥ 80%

Minimal effectiveness: KD60 ≥ 75% or functional mortality ≥ 50%

While the optimal effectiveness is equivalent to the WHOPES evaluation criteria [6], the minimal effectiveness criteria were taken from an unpublished recommendation by WHO (Pierre Guillet).

The physical integrity of the nets was evaluated by applying a proportionate holes index (pHI) based on the number of holes per category and weighted as follows:

pHI = size 1 holes + (size 2 holes × 9) + (size 3 holes × 56) and then a mean hole index calculated for the sample or sub-sample. The multiplication factors were chosen to reflect the approximate surface areas of the hole sizes (4, 36 and 225 cm^2 ^respectively) resulting in one unit of the pHI being equivalent to 4 cm^2 ^of hole surface. Data were then grouped into three categories of pHI 0-24 (or maximum 100 cm^2 ^total hole surface) representing a net in good condition, pHI 25-299 (or maximum 0.1 m^2 ^total hole surface) for a torn net and pHI 300 or above for a severely torn net.

Since previous studies had used a "simple" hole index (sHI) with weights 1, 2, 3 for the hole sizes this index was also calculated in order to allow comparisons:

$$\text{sHI}=\text{size }1\text{ holes}+\left(\text{size }2\text{ holes}\times 2\right)+\left(\text{size }3\text{ holes}\times 3\right)$$

Attrition rate for the net cohort was estimated by adjusting for the potential loss from sampled nets. For each group of sampled nets the number of nets that would have been lost was calculated by applying the observed attrition rates from the remaining nets. These were then added to the actually observed attrition and divided by the total of nets distributed.

The study was conducted according to the principles of the Declaration of Helsinki and the International Guidelines for Ethical Review of Epidemiological Studies and approved by the Uganda Council of Science and Technology.

## Results

### Household and net surveys

Of the 382 households involved in the multi-brand LLIN study in the five villages 211 (55%) had received an Interceptor^® ^LLIN or alpha-cypermethrin conventionally treated net. The proportion of households with only LLIN was 71.6%, those with conventionally treated nets (ITN) only 28.9% and with both 5.2%. The mean number of study nets per household was 1.3 with 25.1% having two and 3.3% three nets. There was no difference between number of nets received between households with only LLIN or only ITN.

The demographic characteristics of the population was not significantly different from the assessment six years earlier [12]. Heads of households were predominantly male with 21.8% female lead households. Average age was 45 years (male 42, female 52), family size 6.1 persons with 1.4 children under five years of age and 1.9 persons per bed or sleeping place. Educational level was better for male than female heads of households with 10.9% and 47.8% being illiterate respectively.

Houses had mainly tin roofs (88.6%), mudded walls (73.0%) and closable windows (91.5%). Fuel for cooking was firewood for 96.2% of households and for almost all families (98.6%) cooking was exclusively outside the main house, usually in a small kitchen hut (assessment during rains in May). A radio was owned by 86.3% and any means of transport by 60.2%, predominantly bicycles (58.3%). The mean wealth score based on assets, animals and land ownership was 27.1 (95% CI 25.9, 28.3) which indicated a statistically significant increase compared to 2000 when it had been 24.4 (23.1, 25.6) using the same methodology.

Demographic and socio-economic variables did not differ statistically between households that had received only LLIN or only ITN (p > 0.2) demonstrating the effectiveness of the random distribution of the study-nets.

**Figure 1:**
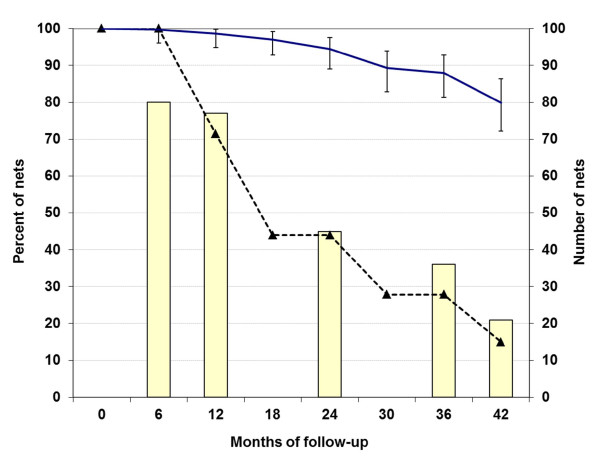
**Sampling and retention of the 280 study nets**. Bars: number of nets sampled, dashed line: proportion of non-sampled nets remaining before sampling, solid line: proportion of non-sampled nets retained with 95% CI.

During the study period a total of 175 of the 190 LLIN and 88 of the 90 ITN were sampled with details shown in Figure 1. In addition, 16 nets (14 LLIN) were lost to follow-up and five remained unsampled. Using the number of lost nets that would have been observed if the sampled nets had been exposed to the same loss rate for each time interval (see methods) the retention rate was calculated and is presented in Figure 1. Looking at the loss rather than retention the attrition rate after one year then was 1.4% (95% CI 0.2, 5.1), after two years 5.6% (2.5, 10.9), three years 12.1% (7.2, 18.7) and after three and a half years 20.1% (13.7, 27.8). The reason for loss was not recorded for all nets but reports from the villages suggest that reasons include nets stolen, burnt and taken to other locations (e.g. boarding school) in addition to being discarded because of damage. The proportion of remaining nets that were assessed in each of the eight net surveys varied between 94.2% and 100%.

### Washing and aspect

Details of the washing frequency are presented in Table 1. During the first 6 months only 33.9% of nets had been washed and the ever-washed proportion increased to 57.0% after one year and 86.5% after two years. Mean number of washes was 0.9 after 12 months and then increased to an average of 1.2 per year after 24 months and 1.4 washes per year after three and 3.5 years respectively. The observed dirtiness of the nets matched the washing pattern with the lowest rate (12.7%) of dirty or very dirty nets found at the six-months inspection and then values fluctuating between 29% and 45% with lower rates corresponding to higher wash frequency in that period (Table 1). Quality control of the reported washing based on the soluble colour marking showed that 97.4% of nets that were reported not washed had the mark present. However, of those washed only in 58.6% the mark was totally gone due to a less than expected washability of the marker, which needed more than one wash to disappear. When in a sub-sample of 78 nets a graded score was applied the mark was at least faded in 80.0% of washed nets.

**Table 1:** Washing of study nets

Indicator	Time of follow-up in months			
	
	6-12	18-24	30-36	37-42
Number of nets in sample	447	237	118	29

Ever washed*	57.0%	86.5%	85.7%	100%
95% CI	49.7, 64.1	78.5, 92.4	74.6, 93.3	76.5, 100

Cumulative washes*				
mean	0.94	2.45	4.27	5.63
95% CI	0.69, 1.18	1-76, 3.14	3.06, 5.27	3.61, 7.34
range	0 to 10	0 to 13	0 to 14	1 to 16

Washed in past 6 months	39.5%	44.3%	57.6%	48.3%
95% CI	35.0, 44.2	37.9, 50.9	48.2, 66.7	29.4, 67.4

Washes in past 6 months				
mean	0.64	0.65	1.01	0.82
95% CI	0.55, 0.75	0.54, 0.77	0.81, 1.23	0.45, 1.21
range	0 to 6	0 to 5	0 to 5	0 to 3

Proportion dirty or very dirty	20.0%	36.5%	28.7%	45.2
95% CI	16.3, 24.0	30.3, 43.0	20.9, 37.6	27.3, 63.9

* refers to end of follow-up period

Nets were generally washed in cold water (96.7%) in a basin (99.5%) and with local soap (95.1%) rather than a detergent and none of the nets was rubbed with or on rocks during washing. Drying occurred outside (98.6%) with about one third (37.3%) lying flat and 62.5% hanging. Washing and drying patterns did not change during the follow-up period and did not differ between the types of net.

### Physical condition

The proportion of nets that were assessed by the field team as having any holes was 19.8% after six months increasing to 33.7% after one year or 25.7% for the six to 12 months observation period presented in Table 2. Wear and tear increased continuously reaching 77.4% of nets with any holes after 36-42 months. Also the proportionate Hole Index (pHI) increased steadily over time with initially 32 pHI units every six months increasing to 69 between three and three and a half years. However, due to the smaller sample at the end of the study the increase in a regression model (general estimation equations) was consistent with a linear increase of 3.8 pHI per month equivalent to 15 cm^2 ^new hole surface area per month or 182 cm^2 ^per year. Adjusting for time of follow-up there was no difference between LLIN and the ITN (p = 0.3). Table 2 also presents the grouped pHI results indicating that after 30-36 months 58% of the nets were still in good condition while 7% were severely torn. This proportion then increased to 13% after 36-42 months but 29% of nets were still in good condition.

**Table 2:** Physical condition of nets during net surveys

Indicator	Time of follow-up in months			
	
	6-12	18-24	30-36	37-42
Number of nets in sample	447	239	122	31

Proportion with any holes	25.7%	46.9%	63.1%	77.4%
95% CI	21.7, 30.0	40.4, 53.4	53.9, 71.7	58.9, 90.4

Proportionate Hole Index (pHI)				
mean	13.4	45.6	91.5	161.4
95% CI	8.8, 18, 0	33.0, 58.2	63.1, 119.9	76.5, 246.3
range	0 to 618	0 to 803	0 to 939	0 to 1220

Simple Hole Index (sHI)				
mean	1.6	4.9	10.3	15.7
95% CI	1.2, 2.0	3.8, 6.0	7.4, 13.2	8.7, 22.8
range	0 to 46	0 to 65	0 to 110	0 to 90

Distribution by pHI category				
0-24	90.6%	72.8%	58.2%	29.0%
25-299	9.2%	23.9%	35.3%	58.1%
300+	0.2%	3.4%	6.6%	12.9%

### Net use

After 6-12 months of use 93.3% of nets were reported to have been used every night. This proportion decreased to 87.1% after 18-24 months, 85.9% after 30-36 months and 81.2% after 36-42 months (p = 0.006). Among the not regularly used nets the proportion of those used not at all during the past six months also increased over time from 20% after 6-12 months to 33%, 56% and 66% in the following observation periods resulting in 12% of all assessed nets not being used at all after 36-42 months. The correlation between non-regular net use and physical condition of the net was even stronger increasing more than three-fold between nets in good condition (6.1% non-regular use) to severely damaged nets (19.0%). This was confirmed by a logit model of non-regular net use which showed a strong gradient for the physical condition with an Odds-ratio of 6.2 (95% CI 2.2, 16.4) for severely damaged compared to nets in good condition after controlling for age of net. In contrast, in the presence of the variable on physical condition the time factor was only relevant in the first 12 months but non-regular use did not increase with age of net thereafter. There was no difference in use between the LLIN and conventionally treated nets controlling for the varying time of observation between them.

### Sampled nets

The average (median) of alpha-cypermethrin content per surface area of the 10 LLIN tested at baseline was 97% of the target dose of 200 mg/m^2 ^with both between and within net variation (Relative Standard Deviation) well below the maximum allowed of 25% for between net variation and of 20% for within net variation [13,14] (see Table 3). Expressed as insecticide per mass of net the value was 6.48 g/kg (Range 5.28, 7.12). For the conventionally treated nets the median alpha-cypermethrin content was also 97% of the target but with considerable between and within net variation of 34% and 28% respectively. All baseline samples had full effectiveness in bio-assays.

**Table 3:** Alpha-cypermethrin content at baseline (10 nets for each type, 3 samples per net), RSD = Relative Standard Deviation; IQR = Inter-Quartile Range

Net type	**Content alpha-cypermethrin in mg/m**^**2**^			Variation of content	
	
	Mean 95% CI	Median IQR Range	Target (median as % of target)	Between net RSD	Within net RSD
LLIN	192.0	194.5	200	11.0%	3.9%
	177, 207	171, 207 146 to 221	(97.3%)		

Conventional	22.0	24.2	24.9	34.3%	27.9%
ITN	17, 27	18, 25 2 to 45	(97.2%)		

**Figure 2:**
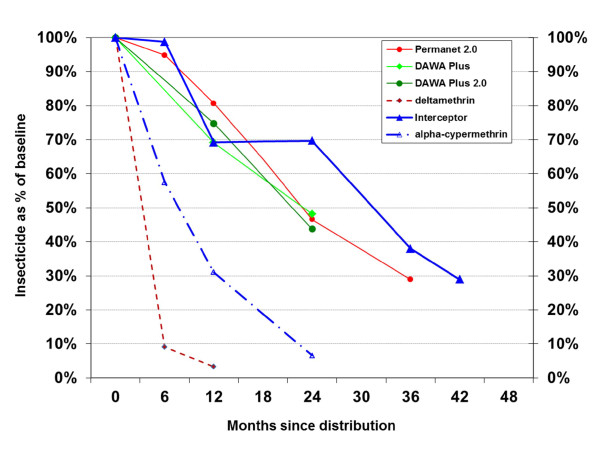
**Decline of active ingredient content of nets as a proportion of the initial dose for the two study nets and three other polyester based LLIN studied in the same setting**.

While at baseline arithmetic mean and median were very close (see Table 3) indicating an approximate normal distribution, the distribution of values from chemical residue tends to deviate from normal with increasing time of follow-up. Therefore, the median alpha-cypermethrin concentration was used for the assessment of the performance of the nets over time (Table 4). After three years of field use the median insecticide concentration for the LLIN was 2.12 g/kg or 73.8 mg/m^2 ^and after three and a half years 1.61 g/kg or 56.2 mg/m^2 ^implying a loss of 62% after three and 71% after 3.5 years respective the initial values. There was some fluctuation of the rate of decline but as shown in Figure 2 the loss rate was very similar to the other polyester LLIN tested in the same villages [12] with approximately 20% per year irrespective of the differing initial doses and deltamethrin being used in the other LLIN brands. After three years 94% (95% CI 81.3, 99.3) of the LLIN samples still had more than 15 mg/m^2 ^alpha-cypermethrin. After three and a half years of field use this figure was 81.0% (58.1, 94.6) and 95.2% (76.2, 99.9) of the LLIN had more than 3 mg/m^2^. In contrast, the median alpha-cypermethrin concentration for the conventional ITN decreased by 69% after 12 months to a median of 7.5 mg/m^2 ^and by 93% after two years with 1.6 mg/m^2^. At the end of year two none of the net samples for the ITN had more than 15 mg/m^2 ^and only 37% more than 3 mg/m^2 ^although the sample was only 8 nets and the confidence interval accordingly very wide (9, 75).

**Table 4:** Alpha-cypermethrin content during follow-up; IQR = Inter-Quartile Range (single sample per net)

Net type	Time of follow-up				
	
	6 months	12 months	24 months	36 months	42 months
**LLIN**					
Number tested	38	37	37	36	21
Median mg/m^2^	193.0	143.0	135.7	73.8	56.2
IQR	166, 210	95, 177	81, 184	51, 105	17, 116
% > 15 mg/m^2^	100%	100%	97%	94%	81%
% > 3 mg/m^2^	100%	100%	97%	94%	95%

**Conventional ITN**					
Number tested	40	40	8		
Median mg/m^2^	13.9	7.5	1.6	Not done	Not done
IQR	2.3, 19.7	0.7, 13, 3	0.8, 7.4		
% > 15 mg/m^2^	40%	18%	0%		
% > 3 mg/m^2^	70%	53%	37%		

Bio-assay results from the WHO cone tests are presented in Table 5. Geometric mean 60-minute knock-down rate for LLIN was above 90% up to three years of follow-up and then dropped to 71%. Mortality rates, however began to fall earlier, 88% after two years and 80% and 68% after three and three and a half years respectively. For the conventional ITN knock down rates stayed surprisingly high at around 95% after two years given the low insecticide concentrations at that time. Mortality was at 66% after two years but the confidence interval was between 47% and 91% given the small sample.

**Table 5:** Bio assay results for *Anopheles gambiae s.s*. expressed as geometric mean

Net type	Time of follow-up				
	
	6 months	12 months	24 months	36 months	42 months
**LLIN**					
Number tested	40	33	37	36	21
KD 60 (95% CI)	99.4% (98.9, 99.8)	97.2% (95.9, 98.4)	93.9% (84.1, 100)	91.9% (86.1, 98.2)	71.4% (47.5, 100)
mortality (95% CI)	98.5% (97.5, 99.4)	97.9% (96.9, 98.9)	87.5% (75.9, 100)	79.5% (72.8, 86.8)	68.3% (54.8, 85.2)

**Conventional ITN**					
Number tested	40	32	8		
KD 60 (95% CI)	91.6% (78.8, 100)	95.0% (93.1, 96.9)	94.8% (88.7, 100)	Not done	Not done
mortality (95% CI)	92.8% (87.0, 98.7)	95.7% (94.1, 97.3)	65.6% (47.3, 91.2)		

Based on the bio-assay results the effectiveness of the tested nets was calculated and results are presented in Table 6. After three years of field use 83% of the LLIN (95% CI 67, 94) still showed optimal performance of either a 60-minute knock-down rate of at least 95% or a 24-hour mortality rate of at least 80%. After three and a half years the optimal effectiveness dropped to 71% (48, 89) but 81% (58, 95) of the LLIN still showed minimal effectiveness of a knock-down rate of at least 75% or a mortality of 50%.

**Table 6:** Optimal and minimal effectiveness of tested nets based on bio-assay results

Net type	Time of follow-up				
	
	6 months	12 months	24 months	36 months	42 months
**LLIN**					
Number tested	40	33	37	36	21
optimal (95% CI)	100% (91.2, 100)	100% (89.5, 100)	94.7% (81.8, 99.3)	83.3% (67.2, 93.6)	71.4% (47.8, 88.7)
minimal (95% CI)	100% (91.2, 100)	100% (89.5, 100)	97.3% (85.8, 99.9)	94.4% (81.3, 99.3)	81.0% (58.1, 94.6)

**Conventional ITN**					
Number tested	40	32	8	Not done	Not done
optimal (95% CI)	95.0% (83.1, 99.4)	100% 89.1, 100)	75% (35, 97)		
minimal (95% CI)	97.5% (86.8, 99.9)	100% 89.1, 100)	100% (63, 100)		

Correlation between bio-assay results and active ingredient content was explored by using optimal effectiveness as "gold standard". Using chemical residue of more than 15 mg/m^2 ^as a test for optimal effectiveness had a sensitivity of 77.2% (95% CI 72, 83) and a specificity of 52.4% (30, 74). Decreasing the cutoff-level to > 3 mg/m^2 ^increased sensitivity to 89.0% (85, 93) but decreased specificity to 38.1% (18, 61). At the proportions of LLIN with optimal effectiveness observed (Table 4) the positive predictive value (ppv) of finding a LLIN sample to have > 3 mg/m^2 ^alpha-cypermethrin was 95.8% (95, 97) at three and 96.5% (96, 98) at three and a half years of follow-up. However, negative predictive values (npv) were only 18.1% (11, 36) and 15.4% (9, 32) respectively.

In order to assess potential factors that influence the performance of the nets a data set was prepared merging the results from chemical residue and bio-assay with information on use, washes, physical condition and aspect (cleanliness) from the net follow-up surveys and information on the household. From the 259 sampled nets complete information on these variables was available for 228 nets, 144 LLIN and 84 ITN. Regression analysis of insecticide content as a function of time of follow-up and the total number of washes the net had received before being sampled showed for the LLIN that each additional wash reduced insecticide content by 4.6 mg/m^2 ^(95% CI 1.8-7.5), i.e. given an average of 1.5 washes per year a loss of 6.9 mg/m^2 ^per year. The total average loss per year was 31.5 mg/m^2 ^(25.0, 37.9) meaning that washing was only responsible for approximately 22% of the annual loss. For the conventional ITN the loss per wash was very similar with 2.1 mg/m^2 ^(0.5, 3.6) or 3.2 mg/m^2 ^per year, but this represented 53% of the overall annual loss of 6.0 mg/m^2 ^(2.0, 10, 0).

A joint analysis of insecticide content using all nets and controlling for net type confirmed the significant negative influence of time (p < 0.0005) and a modest negative impact of washing (p = 0.1). Another factor that could be identified to impact on insecticide content of the net were houses with plastered or brick walls (p = 0.001) which had 21 mg/m^2 ^less insecticide than nets in houses with mudded walls. A marginal influence was seen by nets not used the last 6 months before sampling (p = 0.05) which had 35 mg/m^2 ^higher insecticide levels than regularly used nets, being clean at time of sampling (p = 0.05) which had 12 mg/m^2 ^less than dirty nets but with no differences between level of dirt, the head of household being non-literate (p = 0.8) with 12.3 mg/m^2 ^less insecticide content, and the number of children under five in the household (p = 0.1) indicating that with each additional child the average insecticide content was 4 mg/m^2 ^less. Physical condition showed an association with declining insecticide content initially but this effect disappeared when the interaction term between pHI and time since distribution was introduced (p = 0.07 for interaction). Other interaction terms did not reach significance level. All variables in the model explained 75% of the variability in the data. Variables tested but not included due to lack of significance were wealth quintiles and number of people in the household.

Finally, the possible impact of dirt on the net with respect to bio-assay results was explored. Whether knock-down rate, mortality rate or optimal effectiveness was used as an outcome variable, in all cases no significant impact of the level of dirt on the net was detectable after controlling for insecticide content.

## Discussion

The primary purpose of this study was to assess the field performance of the polyester-based LLIN Interceptor^® ^with respect to the criteria set for WHOPES evaluations. Accordingly, the methodology closely followed the WHOPES guidelines for LLIN testing [6] with the following modifications: i) sample size of 40 nets instead of the recommended 30 per time point was used; ii) at baseline only three instead of the recommended 18 samples were taken per net to establish within-net variability.

Insecticide concentration at baseline for the LLIN was 6.48 g/kg (192.0 mg/m^2^) very close to the value given in the specifications for a 75 denier net of this product of 6.7 g/kg [13]. Between- and within-net variation expressed as relative standard variation was 11.0% and 3.9% respectively, well below the 25% allowed for between net variation and the 20% allowed for within net variation [13,14]. Even when the range of values for the 10 baseline nets was considered (5.3-7.1 g/kg) this was within a ± 25% range of the target dose (5.0-8.4 g/kg). After three years of field use bio-assay results showed that 83.3% of sampled nets had at least a 60-minute knock-down rate of 95% or a 24-hour mortality rate of at least 80% and median alpha-cypermethrin residue was 73.8 mg/m^2 ^or 38% of the initial dose with 94% of net samples having at least 15 mg/m^2^. In contrast, conventionally treated nets lost insecticide content rapidly with only 7% of initial dose left after two years of use (median 1.6 mg/m^2^) and none of the samples having at least 15 mg/m^2 ^although still 75% showed satisfactory bio-assay results. Monitoring net utilization and conditions over the study period demonstrated a tendency of reducing use with increasing deterioration but after three years still 86% of LLIN were regularly used and up to two years (when all conventional ITN were collected) no difference in use rates between LLIN and conventionally treated nets were found suggesting a high level of acceptability of the product. All these findings are in agreement with the conditions set by WHOPES [6] and allow the conclusion that the tested LLIN fulfilled the criteria to be qualified as an LLIN. It must be kept in mind, however, that results may vary from site to site depending on harshness of conditions the nets are used in. With an average washing frequency in the study area of less than two washes per year, a climate with temperatures not exceeding 35°C and reasonable housing conditions this site has to be considered as moderately stressful on the nets. Washing frequencies reported in other studies range between 1.2 and 5.6 per year [15-19] when measured rather than assessed as "intention to wash" [20] and climatic and socio-economic conditions such as those in Western Uganda are found in many places where LLIN are applied. The fact that households in the study area were using LLIN for 8-9 years may have increased the use rate and, hence, the stress on the nets, but also may have resulted in a more careful handling based on passed experiences. This suggests that a similar performance of the Interceptor^® ^LLIN can be expected in a significant proportion of interventions sites were a net culture already exists although it cannot be excluded that the LLIN performs less well in more extreme conditions.

Loss rate of nets (attrition) observed in this study was lower than expected so that sufficient nets were available after 42 months of field use to allow an additional evaluation of field performance. The sample after three and a half years included 21 LLIN so was slightly lower than the recommended 30 [6] but still sufficient to allow a basic statistical analysis. Chemical content results demonstrate a continuous linear decline of insecticide with a median 56.2 mg/m^2 ^alpha-cypermethrin remaining and 81% of samples still giving > 15 mg/m^2 ^(see also Figure 2). Bio-assay results had 71% of LLIN still with optimal biological effectiveness and comparison with results from the preceding year shows a constant rate of decline between year 2-3 and 3-4 of 12% (Table 6). This strongly suggests that protective effectiveness of the LLIN does not dramatically deteriorate the year following the three year cut-off chosen by WHOPES for evaluation purposes although some acceleration of the decline was observed.

Testing of LLIN at the Kyenjojo field site has been going on since the year 2000 and findings of this study regarding socio-economic environment, net use and washing habits do not differ from those previously reported [12]. Also the rate of physical deterioration of the 75 denier polyester Interceptor^® ^LLIN was similar to that found earlier for another 75 denier polyester LLIN product. As shown in Table 2, 63% of nets had any holes after 3 years of follow-up with a mean simple hole index (sHI) of 10.3. In the previously reported studies the respective values had been 79% and 10.7 sHI in the first and 69% and 15.6 sHI in the second [12].

To date three studies on the field performance of Interceptor^® ^have been published. Banek *et al. *[11] studied the LLIN in Liberia using a randomized allocation design of LLIN and conventionally treated nets in a returning refugee setting. Nets were followed for 12 months with six assessments of chemical residue. Mean concentration of alpha-cypermethrin at baseline was 180 mg/m^2 ^(95% CI 152, 208), which gradually declined to 126 mg/m^2 ^(113, 139) after 12 months. This is a 20% loss within the first year and very similar to the finding from Western Uganda. Physical deterioration of the 75 denier LLIN within the first year was also very similar with 26.6% of nets showing any hole in Liberia compared to 25.7% in Uganda.

The other two studies were undertaken in India. Sharma *et al. *[10] studied the Interceptor^® ^LLIN in 19 villages in Odessa State, India and measured net performance with monthly bio-assay tests (WHO cone) using *Anopheles culicifacies *and *Anopheles fluviatilis*. After seven months of regular use by the villagers knock-down rates against the two vectors were 70-80% with 100% mortality on all tested nets. The authors could also demonstrate a significant reduction of vector densities in villages allocated the LLIN compared to untreated or no nets. Dev and co-workers [9] assessed acceptability and side effects in communities in Assam, northeast India. They found that in spite of 9% of users reporting initial and transient effects such as eye irritation acceptability and satisfaction was very high with 80% of users reporting a reduction in visible mosquitoes in the houses. This was confirmed by assessments of vector indoor densities of *Anopheles minimus *which were reduced to zero in the LLIN villages.

Conventionally treated nets in Western Uganda lost insecticide quickly (93% within two years and after approximately three washes) but still had surprisingly high knock-down and mortality rates with 75% of nets still showing optimal performance even at low levels of insecticide. This is, however, in keeping with results reported in the literature for alpha-cypermethrin. Adams *et al *[21] tested low doses of various insecticides in Malawi and demonstrated that even a dose of only 6.5 mg/m^2 ^alpha-cypermethrin resulted in a 93% mortality rate in *Anopheles gambiae s.s*. Similarly high bio-assay results were obtained by Graham *et al *in Pakistan [22] after 21 washes and a target dose of 15 mg/m^2 ^achieving a 49% mortality rate for *Anopheles stephensi*. In The Gambia, Miller *et al *[23] observed a 85% reduction in insecticide content after three washes which is very close to the findings in Uganda. In contrast, Jawara and co-workers [24] found *An. gambiae s.l*. mortality of only 8% after two washes and a target dose of 40 mg/m^2 ^alpha-cypermethrin in The Gambia, but these nets where mainly made of cotton which may have altered the performance.

Considering the consistency of results within the study site in Uganda regarding net use, washing and net performance, variables for similar LLIN products as well as the favorable comparisons with other results for the Interceptor^® ^LLIN and nets conventionally treated with alpha-cypermethrin it appears that the results presented are reliable and valid for the assessment of the performance of the Interceptor^® ^in the environment of Western Uganda.

The second interest of this study was to explore the methodological aspects of determining "useful life" or durability of a given LLIN product. Although insecticide treated nets have been used for over 20 years, this aspect has been very much neglected and only has come into focus of discussions with the scale-up of mass-distribution campaigns for LLIN. While some progress has recently been made to better understand the concept of LLIN durability and how it can be measured [25,26], one of the key issues yet to be solved, is how best to assess the physical condition of the nets. While counting holes in the net has been frequently used to describe the textile integrity [19,27-30], an agreement on how such counts are best, and in a standardized fashion, summarized into a single measure that allows to distinguish between "good" or "serviceable" nets and those that are unlikely to fulfil their protective function, is still lacking.

In this study, a hole index was applied that attempts to improve the previous methodology [12] by applying a weight to the count of three different size categories of holes which is approximately proportionate to the average hole surface area of each hole category. Such an approach has the advantage that the resulting index value for a net corresponds to the total torn net surface and has since been adopted by WHOPES in a slightly modified definition [31]. This value was then used to categorize the nets into good, intermediate and poor physical condition. The cut-offs used are based on the little literature available on this topic and which shows that, if treated with insecticide, nets with holes are still effective [30,32,33] and can provide at least some protection even with a hole surface area of 0.24 m^2 ^[34], which is equivalent to a pHI of 700, i.e. a value more than twice the cut-off used for poor condition in this study.

Categorization of the physical condition of the nets can then be combined with the retention/attrition rates and the results of the bioassay to provide an estimate of the proportion of nets that are still present, in acceptable physical condition and with functional insecticidal protection, hence an assessment of the "useful life" of the LLIN. Applying this approach to the data from this study and assuming that half of the lost nets were thrown away due to wear and tear, would then suggest that 76.9% of the LLIN were fit for use (good or acceptable condition, pHI < 300) and protective after three years and 61.6% after three and a half years. Assuming further that "useful life" is measured by the median survival time of a product in the field, then the Interceptor^® ^LLIN in Western Uganda demonstrated a "useful life" of at least three and a half years and most likely close to four years. There is certainly more work needed to fine-tune cut-offs and definitions, but having a better measure of physical condition of nets, as suggested here, is an important step towards establishing the durability of LLIN.

## Conclusions

In summary, it is concluded that under the conditions in Western Uganda of moderate climate, previous experience in net use and low washing frequency the tested LLIN Interceptor^® ^fulfilled the criteria for phase III of WHOPES evaluations after three years of field use. Based on the obtained data on retention rates, physical condition of nets, the chemical content and bio-assay results and basing outcome evaluation on preliminary criteria, the useful life of this product is estimated to be on average three and a half years. It is also concluded that more widely agreed upon criteria and definitions to evaluate useful life of LLIN are urgently needed.

## Conflict of interest

The authors declare that they have no competing interests.

## Authors' contributions

AK designed the study, undertook the data analysis and drafted the manuscript. WB contributed to the design and implementation in the field and assured data quality, OP was responsible for the chemical analysis results, JG, FA and LK undertook the bio-assay tests and NP participated in the analysis and interpretation of the data and the drafting of the manuscript. All authors contributed to the final version of the text and have read and approved the manuscript.
